# Association between changes in the triglyceride glucose-body roundness Index and cardiovascular disease risk in middle-aged and elderly Chinese adults: a nationwide longitudinal study from 2011 to 2015

**DOI:** 10.3389/fnut.2025.1599601

**Published:** 2025-07-11

**Authors:** Yu Yang, Jinfeng Cao, Jie Lyu

**Affiliations:** ^1^College of Rehabilitation Sciences, Shanghai University of Medicine and Health Sciences, Shanghai, China; ^2^School of Health Science and Engineering, University of Shanghai for Science and Technology, Shanghai, China; ^3^Periodicals Agency of Shanghai University, Shanghai University, Shanghai, China

**Keywords:** cardiovascular disease, triglyceride glucose-body roundness index, middle-aged and elderly, long-term changes, obesity and metabolic

## Abstract

**Background:**

Cardiovascular disease (CVD) is a major global health issue. The triglyceride-glucose (TyG) index, a marker of insulin resistance, and the body roundness index (BRI), reflecting visceral adiposity, are key risk factors for chronic diseases. However, research on the long-term impact of changes in obesity and metabolic markers on CVD risk is limited. This study examines the association between long-term changes in the TyG-BRI composite index and CVD incidence in middle-aged and older Chinese adults.

**Methods:**

Data were obtained from the China Health and Retirement Longitudinal Study, which included 4,446 middle-aged and elderly from 2011 to 2015. The participants were classified into three clusters based on TyG-BRI changes by K-means clustering method. Logistic regression analyses and restricted cubic spline (RCS) regression analyses were used to assess the association between the TyG-BRI and CVD incidence. Receiver operating characteristic (ROC) curves were generated to compare the predictive accuracy of the TyG-BRI, TyG, and BRI.

**Results:**

By the end of 2015, 1,007 participants (22.6%) had developed CVD. The incidence of CVD increased progressively across TyG-BRI clusters. After adjusting for multiple covariates, logistic regression analysis revealed a significant correlation between the TyG-BRI and the onset of CVD [odds ratio, 95% confidence interval: 1.251 (1.139–1.373) per 1 SD increase]. The RCS regression analysis revealed a significant positive and linear relationship between the TyG-BRI and CVD incidence (*P* for overall < 0.001, *P* for nonlinear = 0.874). ROC analysis revealed that the TyG-BRI had greater predictive accuracy for CVD than either BRI or TyG alone did (AUC: 0.678 vs. 0.583 and 0.555, *P* < 0.001).

**Conclusion:**

Long-term variations in the TyG-BRI index are closely associated with CVD risk, demonstrating superior predictive performance compared to using BRI or TyG alone. Our findings offer new insights into the interplay between metabolic dysfunction and cardiovascular risk. TyG-BRI may serve as a more effective auxiliary tool for CVD risk assessment and provides valuable guidance for the early identification of high-risk populations.

## 1 Introduction

Cardiovascular disease (CVD) has become a major global public health concern, with its rising incidence and mortality posing significant threats to healthcare systems and individual health (1, 2). In the context of an aging population, middle-aged and older adults face elevated CVD risk due to prevalent metabolic abnormalities and age-related physiological changes (3, 4). Early identification and intervention targeting metabolic and obesity factors are essential for reducing the CVD burden.

Obesity, especially visceral fat accumulation, is a key contributor to metabolic dysfunction and significantly elevates the risk of CVD (5, 6). Visceral adipose tissue, compared to subcutaneous fat, is more metabolically active and closely linked to insulin resistance (IR), dyslipidemia, and chronic systemic inflammation, all of which drive the development of type 2 diabetes, metabolic syndrome, and atherosclerosis (7–9). Given the significant role of visceral fat in these pathophysiological processes, accurately capturing fat distribution is essential for effective metabolic and cardiovascular risk assessment. However, traditional measures such as body mass index (BMI) inadequately reflect fat distribution and are limited in their predictive power for metabolic diseases and CVD (10). To overcome these limitations, the body roundness index (BRI) was developed, integrating height and waist circumference to better estimate visceral adiposity. Emerging evidence suggests that BRI outperforms traditional anthropometric indices in predicting a range of clinical outcomes, including cardiovascular and metabolic diseases (11–14).

At the same time, IR has been recognized as a key intermediary linking metabolic dysfunction to CVD (7, 8). IR is characterized by impaired responsiveness of peripheral tissues to insulin, and is independently associated with increased morbidity and mortality, particularly in relation to cardiovascular events (15, 16). A previous meta-analysis reported that IR is independently associated with higher CVD risk and all-cause mortality in individuals without diabetes (17). Although the hyperinsulinemia-euglycemic clamp (HEC) technique remains the gold standard for evaluating IR, its invasiveness and high cost limit its utility in large epidemiological studies (18). The triglyceride-glucose index (TyG), a simple and reliable surrogate marker for IR, has been widely adopted in clinical research and practice. It has demonstrated strong predictive capabilities for metabolic syndrome, hypertension, atherosclerosis, and cardiovascular events (19–21). Notably, obesity and IR are intricately interconnected: visceral adiposity promotes IR through chronic inflammation and endoplasmic reticulum stress, while IR exacerbates adiposity by disrupting metabolic homeostasis (22). Recognizing this bidirectional relationship, researchers have explored combining metabolic and obesity-related indices to enhance CVD risk prediction. Composite indices such as triglyceride Glucose-body mass index (TyG-BMI) and triglyceride glucose-waist to hip ratio (TyG-WHtR) have shown superior predictive performance compared to individual markers alone (23). Building on this, recent research has proposed the triglyceride-glucose–body roundness index (TyG-BRI) as a novel composite marker for the assessment of stroke (24). This index integrates both metabolic and anthropometric dimensions, potentially offering enhanced predictive performance compared to traditional single indicators. However, considering that both obesity and metabolic parameters are inherently dynamic, the long-term trajectories of TyG-BRI and their associations with CVD risk remain insufficiently explored.

To fill this knowledge gap, we utilized data from the China Health and Retirement Longitudinal Study to investigate the association between cumulative changes in TyG-BRI from 2011 to 2015 and the risk of incident CVD among middle-aged and older adults aged 45 years and above. We hypothesized that the combined use of TyG and BRI would exert a synergistic effect in predicting CVD risk, outperforming the predictive ability of either index alone.

## 2 Materials and methods

### 2.1 Data source and study population

All data used in this study were derived from the CHARLS, a widely recognized longitudinal study focusing on middle-aged and older adults (≥ 45 years) and representing the national population (25). The baseline national survey of CHARLS was conducted from June 2011 to March 2012, involving 17,708 participants. A multi-stage probability sampling method was used for sample selection. In the first stage, a probability proportional to size (PPS) sampling technique was applied to randomly select 150 county-level units from a sampling framework that covered all counties except Tibet. The final sample of 150 counties was distributed across 28 provinces. The sampling was stratified by region, urban or rural area, and GDP per capita (25).

Participants were followed up every 2–3 years, with waves in 2013 (Wave 2), 2015 (Wave 3), 2018 (Wave 4), and 2020 (Wave 5). In each wave, trained interviewers administered standardized health-related questionnaires. Blood samples and physical measurements were collected in Waves 1 and 3. Further details on the CHARLS sampling methods, anthropometric measurements, and blood biomarkers can be found in previous publications (25, 26)

**FIGURE 1:**
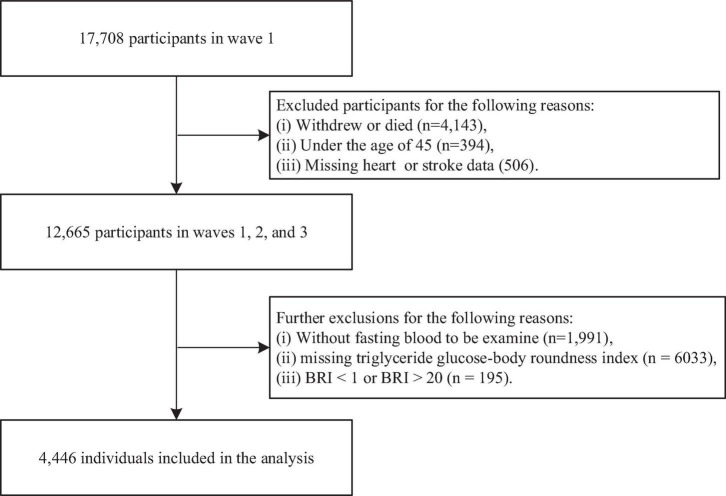
Flowchart for the selection of participants in the study (*n* = 4,446).

This study used data from Waves 1, 2, and 3 of the CHARLS. Initially, 17,708 participants from Wave 1 were included. We excluded 4,143 individuals who dropped out or died by Waves 2 or 3, 394 participants younger than 45 years, and 506 participants with missing data on heart disease or stroke, leaving 12,665 participants. Additional exclusions were made for 1,911 individuals who lacked fasting blood samples, 6,033 participants with missing TyG-BRI data in either Wave 1 or Wave 3, and 195 individuals with BRI values outside the acceptable range (<1 or >20) (27). After these exclusions, the final sample consisted of 4,446 participants (Figure 1).

### 2.2 Data assessment and definitions

#### 2.2.1 Cardiovascular disease

In each CHARLS survey, participants were asked, “Has a doctor ever diagnosed you with stroke/heart disease (including myocardial infarction, coronary heart disease, angina, congestive heart failure, or other heart problems)?” and “Are you currently receiving any of the following treatments (traditional Chinese medicine, Western medicine, other treatment, or none) for stroke/heart disease or its complications?” Following previous studies (28), participants who self-reported a physician-diagnosed heart disease or stroke, or indicated receiving specific treatments for heart disease or stroke, were classified as having CVD.

#### 2.2.2 Triglyceride glucose- body roundness index

The TyG-BRI is derived from multiplying TyG with BRI, as shown in Formula 1. The calculation of BRI is given by Formula 2 (27), while TyG is calculated according to Formula 3 (29).


(1) T⁢y⁢G-B⁢R⁢I=T⁢y⁢G×B⁢R⁢I

(2) $$B\,R\,I=364.2-365.5\,\sqrt{1-\frac{W\,C\,\left(c\,m\right)÷{\left(2\,\pi \right)}^{2}}{{\left(0.5\times H\,e\,i\,g\,h\,t\,\left(c\,m\right)\right)}^{2}}}$$

(3) $$T\,y\,G=l\,n\,\left(\frac{T\,G\,\left(\frac{m\,g}{d\,L}\right)\times F\,B\,G\,\left(\frac{m\,g}{d\,L}\right)}{2}\right)$$

Although data from all three waves were used, only the physical examination data from Waves 1 and 3 contained the necessary indicators for calculating the TyG-BRI. To represent participants’ status over time, a weight of 3 was applied to the indicators from Wave 1 and a weight of 2 was applied to those from Wave 3. All relevant variables, including systolic blood pressure (SBP), diastolic blood pressure (DBP), BRI, glycosylated hemoglobin A1c (HbA1c), fasting blood glucose (FBG), triglycerides (TG), total cholesterol (TC), high-density lipoprotein cholesterol (HDL-c), low-density lipoprotein cholesterol (LDL-c), uric acid, creatinine, and blood urea nitrogen (BUN), were calculated using this weighted approach.

#### 2.2.3 Covariates

In addition to CVD and the TyG-BRI, we collected the following data: (i) demographic information: gender, age, education level, residence, and marital status; (ii) body measurements: SB), DBP, height, weight, and waist circumference; (iii) lifestyle factors: smoking status, drinking status, and exercise habits; (iv) medical history: dyslipidemia, hypertension, diabetes, kidney disease, and liver disease; (v) laboratory examinations: HbA1c, FBG, TG, TC, HDL-c, LDL-c, BUN, uric acid, and creatinine. Supplementary Figure S1 shows the distribution of variables with missing data in our study. To increase accuracy, we updated each measure using all available data from baseline through the end of follow-up. Although the proportion of missing data was small, we employed multiple imputations to address any gaps and maintain a robust sample size.

### 2.3 Statistical analysis

The TyG-BRI categories were defined via the K-means clustering method, which is a widely used unsupervised learning algorithm that partitions a dataset into clusters from proximity to cluster centroids. K-means minimizes the sum of the squared Euclidean distances between data points and their assigned cluster centroids, optimizing cluster formation (30). To determine the optimal number of clusters, we used the elbow method and the gap statistics. The elbow method involves clustering the data with various values of *k* (typically between 1 and 10), calculating the total WSS for each, and plotting the WSS against *k*. The “elbow” point, where the rate of WSS reduction sharply decreases, suggests the ideal number of clusters. The gap statistic further validates the optimal *k* by comparing within-cluster variation for each *k* with that of reference datasets generated under a null hypothesis of a random distribution. The optimal *k* is identified when the gap statistic reaches its maximum, indicating a significant difference between the observed clustering and a random distribution.

Because of the K-means clustering analysis, the study population was divided into three clusters (Cluster 1 to Cluster 3). The characteristics of the participants are presented as the means ± standard deviation (SD) for continuous variables, with group differences assessed via one-way ANOVA. Categorical variables were reported as numbers (percentages) and were analyzed for group differences via Pearson’s chi-square test. To evaluate the association between the TyG-BRI and CVD incidence, univariable and multivariable logistic regression models were developed, with odds ratio (OR) and 95% confidence interval (95% CI). Model 1 was adjusted for age, sex, education, marital status, and residence. Model 2 included adjustments for these variables plus drinking, exercise, HbA1c, TC, HDL-c, LDL-c, uric acid, BUN, creatinine, SBP, and DBP. Model 3 was further adjusted for liver disease, diabetes, and kidney disease in addition to all covariates included in Model 2. Multicollinearity was assessed through variance inflation factors (VIFs) for each variable, all of which were less than 5 (Supplementary Table S1), indicating no significant multicollinearity issues. To assess the effectiveness of the TyG-BRI in predicting CVD incidence, receiver operating characteristic (ROC) curve analysis was conducted via the pROC package. DeLong’s test was applied to compare the predictive performance of the TyG-BRI with that of BRI and TyG. Additionally, a fully adjusted restricted cubic spline (RCS) logistic regression model (using 4 knots at the 5th, 35th, 65th, and 95th percentiles) was employed to examine the linear and dose–response relationships between the TyG-BRI and CVD incidence. Subgroup analyses and interaction tests were conducted to explore potential modifiers. The participants were stratified by age ( < 60 and ≥ 60 years), education level, residence status, marital status, smoking status and drinking status, exercise status, and the presence of conditions such as dyslipidemia, hypertension, diabetes, kidney disease, and liver disease. All the statistical analyses were performed via R software version 4.4.0. A two-tailed *P*-value of < 0.05 was considered statistically significant.

## 3 Results

### 3.1 Clustering of TyG-BRI changes from 2012 to 2015

**FIGURE 2:**
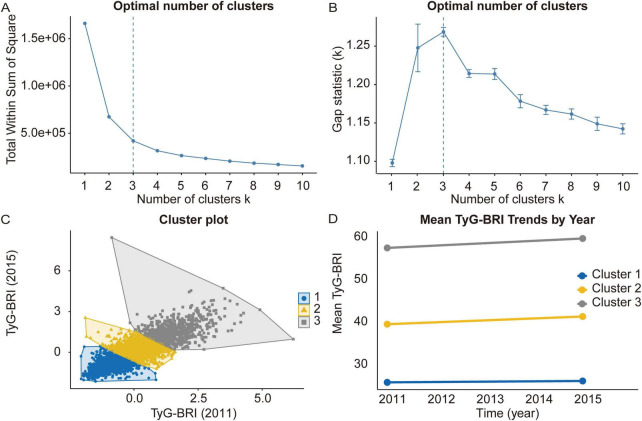
Clustering of the changes in the TyG-BRI from 2011 to 2015. **(A,B)** Determine the optimal number of clusters for the changes in TyG-BRI via the within-cluster sum of squares and gap statistics methods. **(C)** Clustering diagrams for TyG-BRI (2011) and TyG-BRI (2015). **(D)** Data visualization of the mean TyG-BRI across different clusters.

The optimal number of clusters for TyG-BRI variation was determined using the elbow method and gap statistic (Figures 2A,B). Based on the point where the WSS begins to level off and the peak of the gap statistic, the K-means clustering method identified three clusters representing changes in TyG-BRI from 2011 to 2015 (Figure 2C). The ratio of between-cluster sum of squares (BSS) to total sum of squares (TSS) was 74.7%, indicating that the clustering explained a substantial proportion of the total variance and suggested a strong and well-separated structure. As shown in Figure 2D, the mean TyG-BRI values for Cluster 1 (consistently low) were 25.92 in 2011 and 26.24 in 2015. For Cluster 2 (consistently moderate), the values were 39.61 and 41.42, and for Cluster 3 (consistently high), they were 57.58 and 59.80, respectively.

### 3.2 Characteristics of the study participants

Table 1 presents the characteristics of the participants as of 2015, with an average age of 63.04 years, and the 2018 participants (45.39%) were identified as male. Body measurements revealed a significant increase in SBP and DBP as the TyG-BRI score increased. Health history data revealed a marked increase in the incidence of dyslipidemia, hypertension, diabetes, kidney disease, liver disease, and stroke with increasing TyG-BRI. CVD risk follows a similar pattern, with heart disease incidence increasing from 15.9% in Cluster 1 to 28.9% in Cluster 3. The laboratory results further support these trends: HbA1c levels are notably higher in Cluster 3 than in Cluster 1, indicating a potential increase in blood glucose regulation issues with higher TyG-BRI levels. HDL-c levels were inversely related to TyG-BRI scores, with the highest level in Cluster 1 (55.93 mg/dL) and the lowest level in Cluster 3 (44.89 mg/dL). Additionally, BUN and creatinine levels decline progressively across clusters as the TyG-BRI score increases. For further details, Supplementary Table S2 provides a summary of participant characteristics stratified by the presence or absence of CVD.

**TABLE 1:** Characteristics of participants according to changes in TyG-BRI from 2011 to 2015.

Characteristics	Total	Cluster of the TyG-BRI			
		Cluster1	Cluster2	Cluster3	*p*-value
n (%)	4,446	1,746 (39.27)	1,858 (41.79)	842 (18.94)	
**Demographics**					
Age	63.04 ± 8.62	63.31 ± 8.68	62.50 ± 8.64	63.70 ± 8.39	0.001
Gender					< 0.001
Female	2,428 (54.61)	620 (35.5)	1,143 (61.5)	665 (79.0)	
Male	2,018 (45.39)	1,126 (64.5)	715 (38.5)	177 (21.0)	
Education level					< 0.001
Below primary school	2,119 (47.66)	768 (44.0)	868 (46.7)	483 (57.4)	
Primary school	1,005 (22.6)	423 (24.2)	414 (22.3)	168 (20.0)	
Secondary school	900 (20.24)	379 (21.7)	379 (20.4)	142 (16.9)	
High school or above	422 (9.49)	176 (10.1)	197 (10.6)	49 (5.8)	
Residence status					< 0.001
City	1,497 (33.67)	469 (26.9)	699 (37.6)	329 (39.1)	
Rural	2,949 (66.33)	1,277 (73.1)	1,159 (62.4)	513 (60.9)	
Marital status					0.800
Other	495 (11.13)	190 (10.9)	206 (11.1)	99 (11.8)	
Married	3,951 (88.87)	1,556 (89.1)	1,652 (88.9)	743 (88.2)	
**Lifestyle factors**					
Smoking status					< 0.001
No	3,011 (67.72)	908 (52.0)	1,392 (74.9)	711 (84.4)	
Yes	1,435 (32.28)	838 (48.0)	466 (25.1)	131 (15.6)	
Drinking status					< 0.001
No	2,458 (55.29)	789 (45.2)	1,069 (57.5)	600 (71.3)	
Yes	1,988 (44.71)	957 (54.8)	789 (42.5)	242 (28.7)	
Exercise status					0.381
No	1,477 (33.22)	600 (34.4)	598 (32.2)	279 (33.1)	
Yes	2,969 (66.78)	1,146 (65.6)	1,260 (67.8)	563 (66.9)	
**Body measurements**					
SBP	130.63 ± 17.95	126.00 ± 17.15	131.48 ± 17.53	138.34 ± 17.58	< 0.001
DBP	76.07 ± 10.04	73.61 ± 9.78	76.81 ± 9.90	79.52 ± 9.59	< 0.001
BRI	4.36 ± 1.34	3.11 ± 0.53	4.61 ± 0.54	6.38 ± 0.90	< 0.001
**Medical history**					
Dyslipidemia					< 0.001
No	3,453 (77.67)	1,537 (88.0)	1,401 (75.4)	515 (61.2)	
Yes	993 (22.33)	209 (12.0)	457 (24.6)	327 (38.8)	
Hypertension					< 0.001
No	1,434 (32.25)	732 (41.9)	567 (30.5)	135 (16.0)	
Yes	3,012 (67.75)	1,014 (58.1)	1,291 (69.5)	707 (84.0)	
Diabetes					< 0.001
No	3,300 (74.22)	1,469 (84.1)	1,382 (74.4)	449 (53.3)	
Yes	1,146 (25.78)	277 (15.9)	476 (25.6)	393 (46.7)	
Kidney disease					0.649
No	3,976 (89.43)	1,561 (89.4)	1,655 (89.1)	760 (90.3)	
Yes	470 (10.57)	185 (10.6)	203 (10.9)	82 (9.7)	
Liver disease					0.158
No	4,148 (93.3)	1,629 (93.3)	1,745 (93.9)	774 (91.9)	
Yes	298 (6.7)	117 (6.7)	113 (6.1)	68 (8.1)	
CVD					< 0.001
No	3,439 (77.35)	1,436 (82.25)	1,433 (77.13)	570 (67.70)	
Yes	1,007 (22.65)	310 (17.75)	425 (22.87)	272 (32.30)	
Heart disease					< 0.001
No	3,547 (79.78)	1,476 (84.5)	1,482 (79.8)	599 (71.1)	
Yes	899 (20.22)	270 (15.5)	376 (20.2)	243 (28.9)	
Stroke disease					0.024
No	4,266 (95.95)	1,688 (96.7)	1,783 (96.0)	795 (94.4)	
Yes	180 (4.05)	58 (3.3)	75 (4.0)	47 (5.6)	
**Laboratory examinations**					
HbA1c	5.57 ± 0.86	5.39 ± 0.62	5.56 ± 0.82	6.00 ± 1.16	< 0.001
FBG	106.52 ± 29.92	99.82 ± 21.02	106.27 ± 27.77	120.96 ± 42.56	< 0.001
TG	136.29 ± 88.68	97.50 ± 45.41	143.28 ± 79.62	201.31 ± 125.81	< 0.001
TC	191.5 ± 34.89	183.08 ± 32.67	194.60 ± 33.85	202.14 ± 37.53	< 0.001
HDL-c	51.09 ± 12.8	55.93 ± 13.84	49.34 ± 11.12	44.89 ± 10.09	< 0.001
LDL-c	112.16 ± 30.16	107.01 ± 27.83	115.76 ± 29.92	114.87 ± 33.68	< 0.001
Uric acid	4.6 ± 1.19	4.51 ± 1.14	4.60 ± 1.20	4.77 ± 1.24	< 0.001
Creatinine	0.78 ± 0.2	0.81 ± 0.22	0.77 ± 0.18	0.75 ± 0.19	< 0.001
BUN	15.63 ± 3.76	16.10 ± 3.94	15.41 ± 3.66	15.18 ± 3.50	< 0.001

### 3.3 Associations between the TyG-BRI and CVD incidence

**FIGURE 3:**
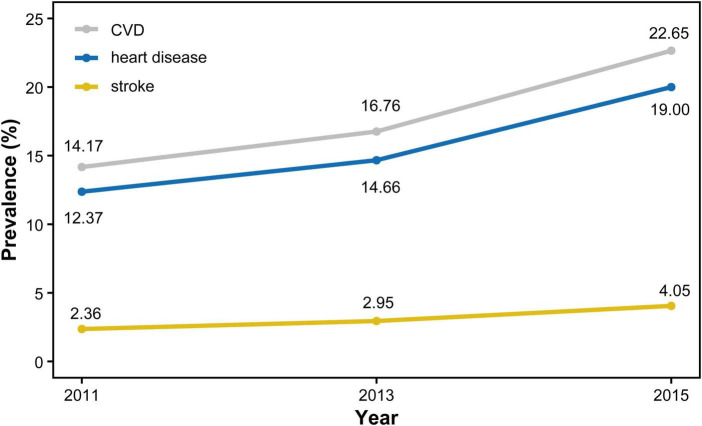
Prevalence of CVD, heart disease and Stroke from 2011 to 2015.

As shown in Figure 3, the prevalence of CVD, heart disease, and stroke increased annually from 2011 to 2015. In 2015, a total of 1,007 participants (22.65%) developed CVD. Additionally, as shown in Table 2, the incidence of CVD increased progressively across TyG-BRI quartiles, from Cluster 1 to Cluster 3, with 310 cases (17.75%) in Cluster 1,425 cases (22.87%) in Cluster 2, and 272 cases (32.30%) in Cluster 3.

**TABLE 2:** Association between the TyG-BRI and CVD.

TyG-BRI	Clusters				Continuous
	Cluster1	Cluster2	Cluster3	P for trend	Per 1 SD increase
Cases, n (%)	310 (17.75)	425 (22.87)	272 (32.30)	–	
Crude, OR (95% CI)	Reference	1.374(1.166–1.618)	2.210(1.829–2.672)	<0.001	1.363(1.273–1.460)
Model 1, OR (95% CI)	Reference	1.376(1.16–1.633)	2.157(1.759–2.647)	<0.001	1.352(1.256–1.456)
Model 2, OR (96% CI)	Reference	1.244(1.034–1.496)	1.754(1.378–2.232)	<0.001	1.277(1.167–1.397)
Model 3, OR (97% CI)	Reference	1.224(1.014–1.478)	1.703(1.330–2.182)	<0.001	1.251(1.139–1.373)

Model 1, adjusted for age, gender, education level, residence status, marital status; Model 2, adjusted for age, gender, education level, residence status, marital status, drinking status, exercise status, SBP, DBP, HbA1c, TC, HDL-c, LDL-c, BUN, uric acid, creatinine; Model 3, adjusted for variables included in Model 2 and diabetes, kidney disease, liver disease. OR, odds ratio; CI, confidence interval; SD, standard deviation.

**FIGURE 4:**
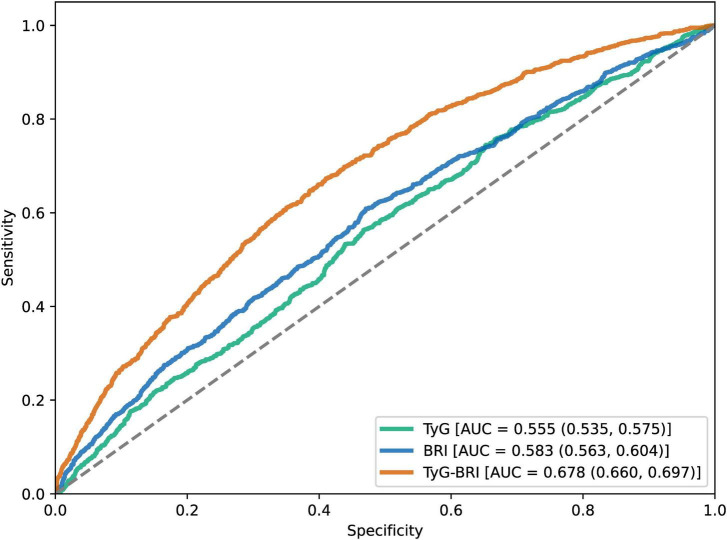
ROC curves for the prediction of CVD incidence based on the TyG-BRI, BRI and TyG.

After adjusting for multiple covariates, the fully adjusted logistic regression model revealed that participants with higher TyG-BRI (Clusters 2 and 3) had an increased OR for CVD events compared with those in Cluster 1. Specifically, the ORs for CVD events were 1.224 (95% CI: 1.014–1.478) for Cluster 2 and 1.703 (95% CI: 1.33–2.182) for Cluster 3. Consistently, when considered a continuous variable, each one-standard-deviation increase in the TyG-BRI was significantly associated with a greater likelihood of CVD events [OR, 95% CI: 1.251 (1.139–1.373)]. Furthermore, logistic regression analysis was used to examine the relationships between the TyG-BRI and specific CVD components (heart disease or stroke). The results revealed a significant association between the TyG-BRI and heart disease incidence [OR, 95% CI: 1.22 (1.001—-1.487) for Cluster 2; OR, 95% CI: 1.727 (1.335–2.234) for Cluster 3; OR, 95% CI: 1.258 (1.142–1.386) per 1 SD increase] but no significant association with stroke. These results are detailed in Supplementary Tables S3, S4. In addition, Figure 4 shows that ROC analysis indicated that the TyG-BRI had significantly greater predictive accuracy for hypertension than either BRI or TyG did (AUC, 0.678 vs. 0.583, *P* < 0.001; AUC, 0.678 vs. 0.555, *P* < 0.001).

**FIGURE 5:**
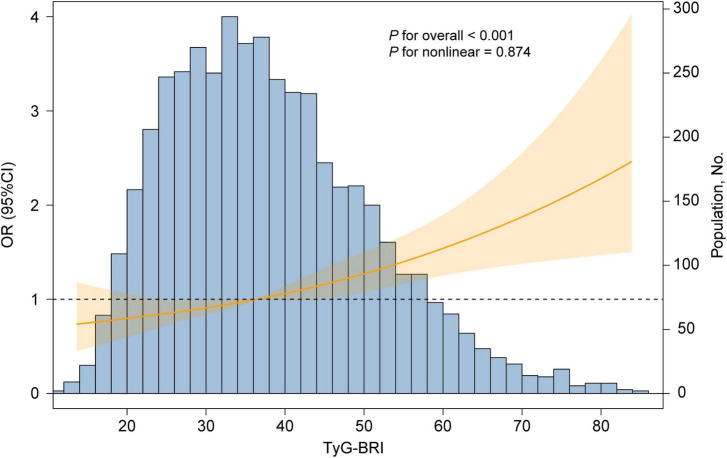
Dose-responsive relationship of the TyG-BRI with the risk of CVD. The model was adjusted for age, gender, education level, marital status, residence, drinking status, exercise status, HbA1c, TC, HDL-c, LDL-c, uric acid, BUN, SBP, DBP, creatinine, liver disease, diabetes and kidney disease.

**FIGURE 6:**
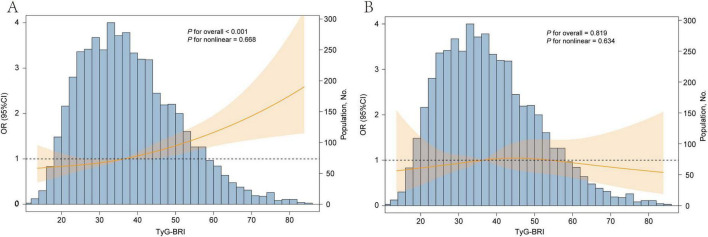
Dose-responsive relationship of the TyG-BRI with the risk of heart disease **(A)** and stroke disease **(B)**. The model was adjusted for age, gender, education level, marital status, residence, drinking status, exercise status, HbA1c, TC, HDL-c, LDL-c, uric acid, BUN, SBP, DBP, creatinine, liver disease, diabetes, kidney disease.

A fully adjusted RCS regression model revealed a positive linear association between the TyG-BRI and CVD incidence (*P* for overall < 0.001, *P* for nonlinear = 0.894) (Figure 5). The RCS model also revealed a linear relationship between the TyG-BRI score and heart disease incidence (*P* for overall < 0.001, *P* for nonlinear = 0.668), whereas no significant association was found between the TyG-BRI and stroke events (*P* for overall = 0.819) (Figure 6).

### 3.4 Subgroup analysis

**FIGURE 7:**
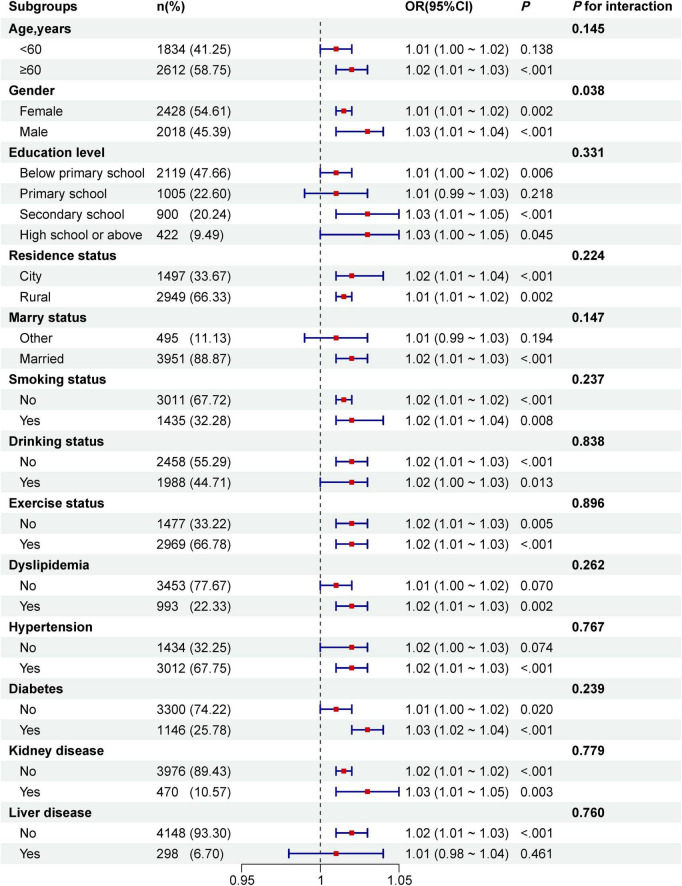
Subgroup analyses of the associations between TyG-BRI and CVD incidence. The model was adjusted for age, gender, education level, marital status, residence, drinking status, exercise status, HbA1c, TC, HDL-c, LDL-c, uric acid, BUN, SBP, DBP, creatinine, liver disease, diabetes, and, kidney disease (excluding the variable for subgroup stratification).

To further investigate the relationship between the TyG-BRI and CVD incidence, we conducted a series of subgroup analyses. As shown in Figure 7, variables such as age, education level, residence status, marital status, smoking and drinking habits, exercise status, and the presence of dyslipidemia, hypertension, diabetes, kidney disease, or liver disease did not significantly affect the associations between the TyG-BRI and CVD incidence (all interactions *P* > 0.05).

However, a significant interaction effect was observed based on gender (*P* for interaction = 0.038). Specifically, for females, the OR for the association between the TyG-BRI and CVD incidence was 1.01 (95% CI: 1.01–1.02, *P* = 0.002). For males, the OR was slightly greater at 1.03 (95% CI: 1.01–1.04, *P* < 0.001). This gender-based difference suggests that the TyG-BRI may be associated with a slightly elevated risk of CVD in males compared with females.

## 4 Discussion

In this nationwide longitudinal study, we observed a significant increase in the prevalence of CVD among middle-aged and elderly Chinese adults from 2011 to 2015. Specifically, a long-term elevated TyG-BRI was associated with a higher incidence of CVD events. Moreover, the predictive ability of TyG-BRI for CVD risk was superior to that of TyG or BRI alone. To the best of our knowledge, this is the first study to assess the trend of the long-term changes in TyG-BRI and its association with CVD risk in this specific population. Our findings may provide novel insights and contribute to the development of more effective strategies for CVD prevention.

Previous studies have established TyG as a strong biomarker for IR and CVD risk (31). Our results are consistent with these findings. For example, the Vascular Metabolism CUN cohort study showed that TyG significantly improved the predictive accuracy of the Framingham coronary heart disease risk model (32) Similarly, Cui et al. reported that the cumulative TyG reflects long-term metabolic burden and is closely associated with CVD risk (33). From a mechanistic perspective, IR contributes to atherosclerosis through multiple biological pathways. IR can lead to endothelial dysfunction, stimulate the migration and proliferation of vascular smooth muscle cells, and activate chronic inflammation and oxidative stress—changes that play a central role in plaque formation and instability (34, 35). IR also impairs insulin-mediated nitric oxide (NO) synthesis by inhibiting the PI3K/Akt signaling pathway, weakening vasodilation and increasing vascular tone, which enhances vasoconstriction (36). In addition, IR is associated with elevated aldosterone levels, which activate epithelial sodium channels (ENaC), increase sodium permeability, and contribute to vascular stiffness and hypertension (37). IR is also linked to a prothrombotic state through increased platelet activation, upregulation of coagulation factors, and reduced fibrinolysis, raising the risk of thrombotic events (38).A high TyG, as a reliable indicator of IR, has been shown to be associated with various CVD risk factors, including metabolic syndrome, coronary artery disease, and heart failure (39–41). These mechanisms collectively accelerate the development and progression of CVD.

Obesity contributes to adipose tissue dysfunction. Imbalanced adipokine secretion leads to chronic systemic inflammation, which underlies the onset and progression of cardiovascular complications (42). BRI, based on an ellipse model of body shape, estimates visceral and total body fat percentages using eccentricity (27). Emerging evidence suggests that BRI better reflects fat distribution than traditional body fat measures. This makes BRI a strong predictor of metabolic disorders. In our study, we found that cumulative BRI was significantly higher in individuals with CVD than in those without (4.68 ± 1.44 vs. 4.26 ± 1.29, *P* < 0.001). Data from the National Health and Nutrition Examination Survey (NHANES) and the CHARLS have shown that higher BRI is significantly linked to increased CVD risk (43, 44). One large cohort study of 15,848 individuals with diabetes or prediabetes found a nonlinear relationship between BRI and cardiovascular mortality over 8 years of follow-up. When BRI exceeded 5.21, CVD risk increased by 13% (45). Similarly, the Tehran Lipid and Glucose Study followed 6,840 women for up to 16 years and found that higher BRI was associated with a 60% increase in CVD risk in postmenopausal women or those who transitioned into menopause during the study period (46). The mechanisms linking BRI to CVD may involve visceral fat accumulation, which triggers a series of adverse effects including increased oxidative stress, higher levels of pro-inflammatory cytokines, disrupted adipokine secretion, hypoxia, and IR. These changes collectively impair cardiovascular structure and function, ultimately promoting the development of CVD (47–49).

Our study advances this field by integrating TyG and BRI into a combined indicator—TyG-BRI—which captures both metabolic status and obesity-related risk. This dual biomarker showed stronger associations with CVD risk compared to either component alone. These findings are consistent with other combined indices like TyG-BMI, TyG-WC, and TyG-WHtR, which have also shown significant associations with chronic disease outcomes (50, 51). For example, TyG-BMI has been linked to the severity of coronary artery lesions (52), and is useful in predicting major adverse cardiovascular events in patients undergoing percutaneous coronary intervention (51, 53). It is important to note that metabolic and obesity-related markers, such as TyG index, BMI, and BRI are not static but fluctuate over time due to factors such as aging, lifestyle changes, and disease progression. We applied the K-means clustering method to categorize individuals into low, medium, and high clusters based on the changes in their TyG-BRI from 2011 to 2015. The results showed that the average TyG-BRI levels in all three groups increased over time, and the prevalence of cardiovascular diseases (including stroke and heart disease) also rose annually. This suggests that the dynamic changes in the TyG-BRI, as a composite metabolic index, may have strong predictive value for cardiovascular health. These findings align with results from a study based on CHARLS. That study also observed a continuous increase in BRI over years, particularly in women, the elderly, and urban residents, with the increase being more pronounced in these groups. Moreover, prolonged high BRI levels were closely associated with the occurrence of stroke and heart disease events (54). Additionally, another study explored the temporal relationship between BMI and TyG and found that changes in BMI often preceded changes in TyG (55). This suggests that the worsening of obesity may serve as a precursor to metabolic abnormalities, which may already have a potential impact on cardiovascular health at earlier stages. In fact, these markers can be seen as a “metabolic ledger” that records an individual’s long-term metabolic burden. If this ledger remains in a “high debt” state for an extended period, cardiovascular damage will gradually accumulate and intensify unnoticed. From a physiological mechanism perspective, the impact of metabolic abnormalities on CVD is a gradual process. During this process, the function of adipose tissue is progressively impaired, leading to an increase in pro-inflammatory factors (such as TNF-α and IL-6) and a decrease in anti-inflammatory factors (such as adiponectin), which contributes to a state of chronic low-grade inflammation (56, 57). This inflammatory response not only exacerbates IR but also promotes the accumulation of visceral fat, further worsening metabolic disturbances and creating a vicious cycle that drives the development of CVD.

Although the AUC for TyG-BRI in our study was 0.678—higher than that of BRI (0.583) and TyG (0.555)—its discriminatory power is still considered moderate. This is like findings from other studies. For example, data from the Isfahan Cohort Study showed that TyG had AUC values of 0.631 for myocardial infarction, 0.594 for unstable angina, 0.611 for overall CVD, and 0.595 for stroke (58). In the U.S. population, TyG-WC and TyG-WHtR were more effective in predicting CVD and mortality, with AUCs of 0.628 and 0.614, respectively (23). They also showed moderate diagnostic accuracy for coronary artery disease, congestive heart failure, and myocardial infarction (AUCs of 0.654 and 0.642). These findings suggest that while combined indices improve predictive performance, their independent value is still limited. Therefore, they should be used alongside other clinical indicators rather than on their own.

In addition, when further exploring the associations between the TyG-BRI score and specific CVD components, we identified a significant correlation with heart disease, which is consistent with the findings of previous studies on TyG-related indices (59). However, no significant association was found between the TyG-BRI and stroke incidence, which is inconsistent with the findings of several earlier studies (60). The small number of stroke cases may have limited our ability to observe this association.

To further explore and elucidate the differences and characteristics of our findings across different subgroups, we conducted subgroup analyses, which revealed the distinct predictive power of the TyG-BRI between males and females. Males presented a greater CVD risk with an elevated TyG-BRI. This gender difference can be explained by several physiological and metabolic mechanisms. Studies have shown that men and women differ significantly in fat distribution, hormonal levels, and metabolic characteristics. Men are more likely to accumulate visceral fat, which is closely associated with higher risks of metabolic diseases, whereas women tend to accumulate more subcutaneous fat, particularly in the hips and thighs (61, 62). Furthermore, higher testosterone levels in men are linked to IR and an increased incidence of glucose and lipid metabolism abnormalities, which further increase their risk of metabolic diseases (63). These gender differences not only suggest the potential for gender-specific applications of the TyG-BRI but also offer directions for future research aimed at understanding and utilizing the TyG-BRI for personalized CVD and metabolic disease risk assessment.

Despite the important findings of our study, several limitations should be acknowledged. First, this was an observational study. To capture the long-term changes in both CVD and the TyG-BRI over time, we did not exclude individuals who had already been diagnosed with CVD prior to the blood tests. As a result, the causal relationship between TyG-BRI and CVD incidence should be interpreted with caution. Second, health history data were based on self-reports, which may introduce recall bias. Finally, our participants were all middle-aged or older Chinese individuals, and further research is needed to validate whether our findings apply to individuals from different countries and across a broader age range.

## 5 Conclusion

Long-term changes in the TyG-BRI index are strongly associated with an increased risk of CVD, and this composite indicator demonstrates superior predictive power compared to TyG or BRI alone. As a comprehensive marker that simultaneously reflects metabolic dysfunction and obesity status, TyG-BRI serves as a more effective auxiliary tool for CVD risk assessment. It offers valuable insights for the early identification of high-risk populations. Moreover, given the global rise in obesity rates, integrating TyG-BRI into public health strategies could enable more accurate risk stratification and improve the prevention and management of obesity-related diseases.

## Data Availability

Publicly available datasets were analyzed in this study. This data can be found at: The datasets generated and/or analyzed during the current study are available in the China Health and Retirement Longitudinal Study repository (http://charls.pku.edu.cn).
