# Supplementary material for: Association between changes in the triglyceride glucose-body roundness Index and cardiovascular disease risk in middle-aged and elderly Chinese adults: a nationwide longitudinal study from 2011 to 2015
Source: Front Nutr. 2025 Jul 11;12:1560617. doi: 10.3389/fnut.2025.1599601 (PMC12289664; doi:10.3389/fnut.2025.1599601)
Supplement: Supplementary file 1 [file Table_1.docx]

| Table S1. Collinearity Statistics. | | | |
| --- | --- | --- | --- |
|  | GVIF | Df | GVIF^(1/(2*Df)) |
| TyG-BRI | 1.668236 | 2 | 1.136487 |
| Age | 1.587976 | 1 | 1.260149 |
| Education level | 1.366771 | 3 | 1.053455 |
| Marry status | 1.12687 | 1 | 1.061541 |
| Residence status | 1.083032 | 1 | 1.040688 |
| Gender | 2.071259 | 1 | 1.439187 |
| Drinking | 1.328099 | 1 | 1.152432 |
| Exercise status | 1.005304 | 1 | 1.002649 |
| HbA1c | 1.39014 | 1 | 1.179042 |
| TC | 3.587887 | 1 | 1.894172 |
| HDL-c | 1.329076 | 1 | 1.152856 |
| LDL-c | 3.218131 | 1 | 1.793915 |
| Uric acid | 1.575185 | 1 | 1.255064 |
| BUN | 1.226803 | 1 | 1.107611 |
| Creatinine | 1.738471 | 1 | 1.318511 |
| SBP | 2.261782 | 1 | 1.503922 |
| DBP | 2.170579 | 1 | 1.473288 |
| Liver disease | 1.023764 | 1 | 1.011812 |
| Diabetes | 1.38184 | 1 | 1.175517 |
| Kidney | 1.049943 | 1 | 1.024667 |

| Table S2. Characteristics of individuals classified by CVD. | | | | | |
| --- | --- | --- | --- | --- | --- |
| Characteristics | |  | CVD | | p-value |
|  |  | Total | No | Yes |  |
| n (%) | | 4446 | 3439 (77.4) | 1007 (22.6) |  |
| **Demographics** | |  |  |  |  |
| Age | | 63.04 ± 8.62 | 62.48 ± 8.63 | 64.98 ± 8.32 | <0.001 |
| Gender | |  |  |  |  |
|  | Female | 2428 (54.61) | 1843 (53.6) | 585 (58.1) | 0.013 |
|  | Male | 2018 (45.39) | 1596 (46.4) | 422 (41.9) |  |
| Education level | |  |  |  |  |
|  | Below primary school | 2119 (47.66) | 1624 (47.2) | 495 (49.2) | 0.225 |
|  | Primary school | 1005 (22.6) | 780 (22.7) | 225 (22.3) |  |
|  | Secondary school | 900 (20.24) | 717 (20.8) | 183 (18.2) |  |
|  | High school or above | 422 (9.49) | 318 (9.2) | 104 (10.3) |  |
| Residence status | |  |  |  |  |
|  | City | 1497 (33.67) | 1136 (33.0) | 361 (35.8) | 0.104 |
|  | Rural | 2949 (66.33) | 2303 (67.0) | 646 (64.2) |  |
| Marry status | |  |  |  |  |
|  | Other | 495 (11.13) | 349 (10.1) | 146 (14.5) | <0.001 |
|  | Married | 3951 (88.87) | 3090 (89.9) | 861 (85.5) |  |
| **Lifestyle factors** | |  |  |  |  |
| Smoking status | |  |  |  |  |
|  | No | 3011 (67.72) | 2287 (66.5) | 724 (71.9) | 0.001 |
|  | Yes | 1435 (32.28) | 1152 (33.5) | 283 (28.1) |  |
| Drinking status | |  |  |  |  |
|  | No | 2458 (55.29) | 1833 (53.3) | 625 (62.1) | <0.001 |
|  | Yes | 1988 (44.71) | 1606 (46.7) | 382 (37.9) |  |
| Exercise status | |  |  |  |  |
|  | No | 1477 (33.22) | 1122 (32.6) | 355 (35.3) | 0.129 |
|  | Yes | 2969 (66.78) | 2317 (67.4) | 652 (64.7) |  |
| **Body measurements** | |  |  |  |  |
| SBP | | 130.63 ± 17.95 | 129.49 ± 17.70 | 134.51 ± 18.26 | <0.001 |
| DBP | | 76.07 ± 10.04 | 75.73 ± 9.99 | 77.23 ± 10.12 | <0.001 |
| BRI | | 4.36 ± 1.34 | 4.26 ± 1.29 | 4.68 ± 1.44 | <0.001 |
| **Medical history** | |  |  |  |  |
| Dyslipidemia | |  |  |  |  |
|  | No | 3453 (77.67) | 2848 (82.8) | 605 (60.1) | <0.001 |
|  | Yes | 993 (22.33) | 591 (17.2) | 402 (39.9) |  |
| Hypertension | |  |  |  |  |
|  | No | 1434 (32.25) | 1219 (35.4) | 215 (21.4) | <0.001 |
|  | Yes | 3012 (67.75) | 2220 (64.6) | 792 (78.6) |  |
| Diabetes | |  |  |  |  |
|  | No | 3300 (74.22) | 2620 (76.2) | 680 (67.5) | <0.001 |
|  | Yes | 1146 (25.78) | 819 (23.8) | 327 (32.5) |  |
| Kidney disease | |  |  |  |  |
|  | No | 3976 (89.43) | 3156 (91.8) | 820 (81.4) | <0.001 |
|  | Yes | 470 (10.57) | 283 (8.2) | 187 (18.6) |  |
|  |  |  |  |  |  |
| Liver disease | |  |  |  |  |
|  | No | 4148 (93.3) | 3260 (94.8) | 888 (88.2) | <0.001 |
|  | Yes | 298 (6.7) | 179 (5.2) | 119 (11.8) |  |
| **Laboratory examinations** | | |  |  |  |
| HbA1c | | 5.57 ± 0.86 | 5.56 ± 0.87 | 5.63 ± 0.81 | 0.013 |
| FBG | | 106.52 ± 29.92 | 106.13 ± 30.19 | 107.87 ± 28.97 | 0.104 |
| TG | | 136.29 ± 88.68 | 133.37 ± 88.38 | 146.28 ± 89.05 | <0.001 |
| TC | | 191.5 ± 34.89 | 191.34 ± 34.56 | 192.04 ± 36.02 | 0.575 |
| HDL-c | | 51.09 ± 12.8 | 51.52 ± 12.76 | 49.61 ± 12.86 | <0.001 |
| LDL-c | | 112.16 ± 30.16 | 112.08 ± 29.70 | 112.42 ± 31.70 | 0.756 |
| Uric acid | | 4.6 ± 1.19 | 4.57 ± 1.18 | 4.68 ± 1.23 | 0.015 |
| Creatinine | | 0.78 ± 0.2 | 0.78 ± 0.20 | 0.79 ± 0.21 | 0.106 |
| BUN | | 15.63 ± 3.76 | 15.64 ± 3.71 | 15.60 ± 3.93 | 0.754 |
| Note: Variables are presented as mean ± SD or n (%). Abbreviations: SBP, systolic blood pressure; DBP, diastolic blood pressure; BRI, body roundness index; HbA1c, glycosylated hemoglobin A1c; FBG, fasting blood glucose; TG, triglyceride; TC, total cholesterol; HDL‐c, high‐density lipoprotein cholesterol; LDL-c, low-density lipoprotein cholesterol; BUN, blood urea nitrogen; TyG-BMI, triglyceride glucose-body roundness index. | | | | | |

| Table S3. Association between the TyG-BRI and heart disease. | | | | |  |
| --- | --- | --- | --- | --- | --- |
| TyG-BRI | Clusters | | | | Continuous |
|  | Cluster1 | Cluster2 | Cluster3 | P for trend | Per 1 SD increase |
| Cases, n (%) | 270 (15.5) | 376 (20.2) | 243 (28.9) | - |  |
| Crude, OR (95% CI) | Reference | 1.387(1.168-1.648) | 2.218(1.820-2.702) | <0.001 | 1.365(1.271-1.465) |
| Model 1, OR (95% CI) | Reference | 1.352(1.129-1.618) | 2.094(1.692-2.591) | <0.001 | 1.336(1.237-1.443) |
| Model 2, OR (96% CI) | Reference | 1.240(1.022-1.506) | 1.771(1.375-2.279) | <0.001 | 1.283(1.168-1.410) |
| Model 3, OR (97% CI) | Reference | 1.220(1.001-1.487) | 1.727(1.335-2.234) | <0.001 | 1.258(1.142-1.386) |
| Model 1, adjusted for age, gender, education level, residence status, marry status ;  Model 2, adjusted for age, gender, education level, residence status, marry status, drinking status, exercise status, SBP, DBP, HbA1c, TC, HDL-c, LDL-c, BUN, uric acid, creatinine;  Model 3, adjusted for variables included in Model 2 and diabetes, kidney disease, liver disease. OR, odds ratio; CI, confidence interval; SD, standard deviation | | | | | |

| Table S4. Association between the TyG-BRI and stroke disease. | | | | |  |
| --- | --- | --- | --- | --- | --- |
| TyG-BRI | Clusters | | | | Continuous |
|  | Cluster1 | Cluster2 | Cluster3 | P for trend | Per 1 SD increase |
| Cases, n (%) | 58 (3.3) | 75 (4.0) | 47 (5.6) | - |  |
| Crude, OR (95% CI) | Reference | 1.224(0.863-1.736) | 1.721(1.161-2.551) | 0.009 | 1.206(1.047-1.388) |
| Model 1, OR (95% CI) | Reference | 1.487(1.034-2.137) | 2.270(1.479-3.484) | <0.001 | 1.335(1.147-1.554) |
| Model 2, OR (96% CI) | Reference | 1.122(0.758-1.660) | 1.244(0.746-2.076) | 0.537 | 1.062(0.878-1.283) |
| Model 3, OR (97% CI) | Reference | 1.082(0.728-1.610) | 1.158(0.689-1.949) | 0.912 | 1.011(0.833-1.227) |
| Model 1, adjusted for age, gender, education level, residence status, marry status ;  Model 2, adjusted for age, gender, education level, residence status, marry status, drinking status, exercise status, SBP, DBP, HbA1c, TC, HDL-c, LDL-c, BUN, uric acid, creatinine;  Model 3, adjusted for variables included in Model 2 and diabetes, kidney disease, liver disease. OR, odds ratio; CI, confidence interval; SD, standard deviation | | | | | |
