# Supplementary figures and images for: Association between changes in the triglyceride glucose-body roundness Index and cardiovascular disease risk in middle-aged and elderly Chinese adults: a nationwide longitudinal study from 2011 to 2015
Source: Front Nutr. 2025 Jul 11;12:1560617. doi: 10.3389/fnut.2025.1599601 (PMC12289664; doi:10.3389/fnut.2025.1599601)

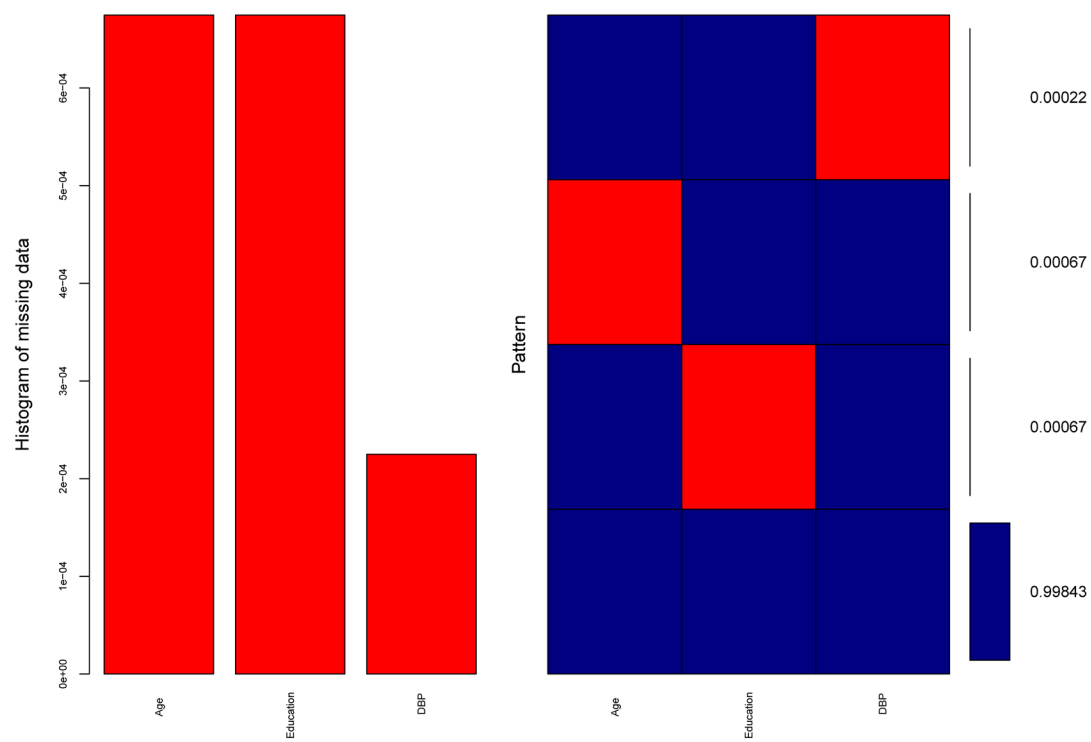

**Figure S1.** Distribution of variables with missing data.

Supplement: Supplementary file 2 [file Image_1.pdf]
